# Zinc dysregulation in cancers and its potential as a therapeutic target

**DOI:** 10.20892/j.issn.2095-3941.2020.0106

**Published:** 2020-08-15

**Authors:** Jie Wang, Huanhuan Zhao, Zhelong Xu, Xinxin Cheng

**Affiliations:** ^1^Department of Physiology and Pathophysiology, Tianjin Medical University, Tianjin 300070, China

**Keywords:** Cancer, cancer therapy, zinc homeostasis, zinc transporter

## Abstract

Zinc is an essential element and serves as a structural or catalytic component in many proteins. Two families of transporters are involved in maintaining cellular zinc homeostasis: the ZIP (SLC39A) family that facilitates zinc influx into the cytoplasm, and the ZnT (SLC30A) family that facilitates zinc efflux from the cytoplasm. Zinc dyshomeostasis caused by the dysfunction of zinc transporters can contribute to the initiation or progression of various cancers, including prostate cancer, breast cancer, and pancreatic cancer. In addition, intracellular zinc fluctuations lead to the disturbance of certain signaling pathways involved in the malignant properties of cancer cells. This review briefly summarizes our current understanding of zinc dyshomeostasis in cancer, and discusses the potential roles of zinc or zinc transporters in cancer therapy.

## Introduction

As an essential trace element, zinc plays crucial roles in protein structure, enzymatic activity, and gene regulation. Over 300 enzymes require zinc for their activities, and more than 2,000 transcription factors require zinc for maintenance of structural integrity and DNA binding activity. Thus, zinc metabolism and homeostasis are regulated in a sophisticated manner for normal cellular functions^1^. Both zinc deficiency and zinc excess may contribute to various health problems, including metabolic diseases, endocrine diseases, neurodegenerative diseases, immune deficiencies, cardiovascular diseases, and cancers^2–5^. In this review, we summarize the recent epidemiological, experimental, and clinical findings of zinc dyshomeostasis in cancer, and outline the potential clinical applications of zinc in cancer prevention, diagnosis, and therapy.

## Zinc signaling

Two forms of zinc exist in our body: protein-bound and free zinc. Protein-bound zinc stabilizes and functionalizes proteins. Proteome analyses indicates that nearly 10% of genome-encoded proteins can bind zinc through interactive regions, especially zinc-finger motifs^6^. Free zinc is also known as labile, chelatable, or mobile zinc. The intracellular free zinc concentration is tightly regulated within the pico- to low nanomolar range. Recently, the role of free zinc as a signaling molecule has received extensive attention.

Zinc signaling can be triggered by transient changes in free zinc at both extracellular and intracellular sites. Extracellular zinc signaling can be initiated by zinc efflux across the plasma membrane by zinc transporters or release from secretory vesicles that accumulate large amounts of zinc. Then, zinc serves as a ligand of several receptor channels on the plasma membrane, such as the zinc sensing receptor (ZnR/GPR39), α-amino-3-hydroxy-5-methyl-4-isoxazolepropionic acid (AMPA) receptors, N-methyl-D-aspartate (NMDA) receptors, voltage-dependent Ca2+ channels (VDCC), and γ-aminobutyric acid_A_ (GABAA) receptors^7^.

Zinc signaling originating from zinc influx, can be divided into two categories depending on the timescale in which it acts^8,9^. Fast zinc signaling occurs within a few seconds to minutes. Zinc serves as an intracellular second messenger and modulates several signaling cascades in both cases. The other zinc signaling pathway, namely “late” zinc signaling, takes place over a longer timescale. In this case, transcriptional regulation of zinc-related proteins, such as ZIPs and ZnTs, is triggered by diverse extracellular stimuli, including cytokines and growth factors. Then, alterations in intracellular zinc homeostasis result in the regulation of downstream molecular targets, including protein kinase C (PKC), cAMP-dependent protein kinase (PKA), Ca/calmodulin-dependent protein kinase II (CaMKII), phosphodiesterases (PDEs), protein tyrosine phosphatases (PTPs), and transcription factors (for example, NF-κB)^10^.

## Regulation of zinc homeostasis

**Figure 1:**
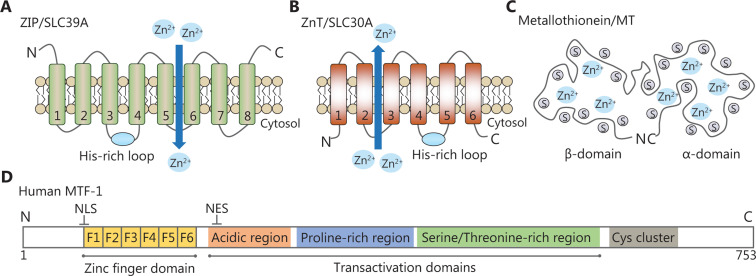
Representative structural domains of zinc-related proteins. (A, B) The predicted topologies of ZIP and ZnT transporters. The His-rich cluster of zinc transporters is indicated by a blue solid circle. (C) Schematic drawing of the metallothionein structure. It contains seven zinc ions bound in two independent domains. (D) Schematic diagram of human MTF-1. It has one zinc finger DNA-binding domain and three transcriptional activation domains. A cysteine cluster is also found at the C-terminus.

**Figure 2:**
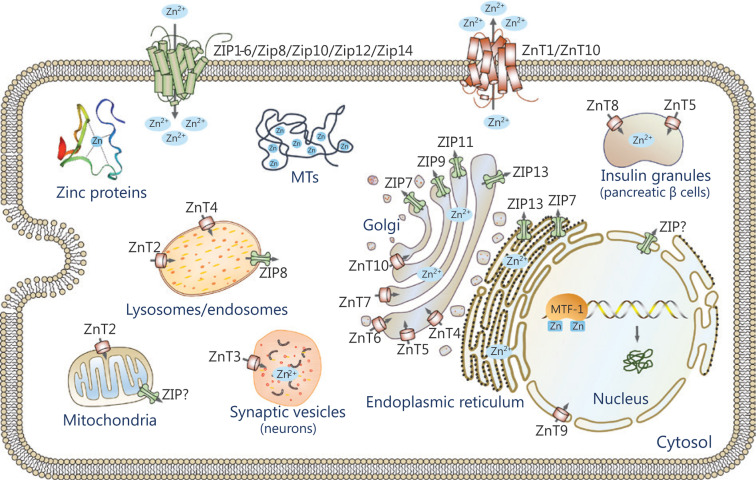
Subcellular localization of zinc transporters. The localizations of ZIPs and ZnTs are shown based on available information. The arrow indicates the direction of zinc mobilization. ZIPs import zinc into the cytoplasm from the extracellular compartments or intracellular organelles, while ZnTs move zinc in the opposite direction.

Zinc homeostasis is maintained primarily by the coordinated actions of zinc transporters, metallothioneins (MTs) and metal-regulatory transcription factor 1 (MTF-1). Mammalian zinc transporters have been classified into two major families, the ZIP (ZRT, IRT-like protein) family, also called solute carrier family 39A (SLC39A), and the ZnT (zinc transporter) family, also called SLC30A proteins^11,12^. The ZIP family comprises 14 members (ZIP1-14) and they facilitate zinc influx into the cytoplasm from the extracellular compartments or intracellular organelles, including the endoplasmic reticulum (ER), mitochondria, and Golgi apparatus. Most ZIP transporters contain eight predicted transmembrane domains (TMDs) with extracellular or luminal N- and C-termini (**Figure 1A**). A histidine-rich cluster in the intracellular loop between TMDs III and IV is thought to be important for zinc binding or regulation. The ZnT family is comprised of 10 members (ZnT1-10) and lowers cytoplasmic zinc in the opposite direction. Most members of this family are predicted to have 6 TMDs with cytoplasmic N- and C-termini (**Figure 1B**), while ZnT5 has an exceptionally long N-terminal region with nine putative TMDs. Similar to the ZIP proteins, most ZnT proteins have a cytoplasmic His-rich loop between TMDs IV and V^13^. Both ZIP and ZnT transporters are localized at the plasma membrane or specific subcellular compartments and display changes in response to various stimuli (**Figure 2**). In addition, they are subject to post-transcriptional or post-translational regulatory mechanisms, including mRNA stability, miRNA regulation, protein cleavage, protein phosphorylation, and protein ubiquitination^14,15^. Diversities in the localization, trafficking and regulatory mechanism of zinc transporters are crucial to maintain cellular zinc homeostasis. Recent studies involving mice and humans have revealed that zinc dysfunction caused by knockout (KO) or mutations of zinc transporters is strongly linked to clinical diseases^11^. We briefly summarize the information in **Supplementary Table S1**.

MTs are cysteine-rich metal-binding proteins with low molecular weights (**Figure 1C**). Humans possess four classes of MT isoforms (MT1, MT2, MT3, and MT4) that can bind up to seven zinc ions to cysteines in two domains. Thus, MTs are capable of regulating intracellular zinc distribution, storage, and release to protect cells against oxidative stresses^16^. MTF-1, a cellular zinc sensor, possesses six Cys2His2 zinc fingers and three transcriptional activation domains (**Figure 1D**). These regions are responsible for zinc sensing and zinc-dependent transcriptional activation. Once activated, MTF-1 translocates to the nucleus and regulates genes that are essential for zinc homeostasis *via* metal response elements (MREs)^17^.

##  Zinc transporters and zinc signaling in cancer

Because zinc regulates a multitude of cellular functions, zinc dyshomeostasis may cause various abnormalities, particularly the initiation or progression of cancer.

## Zinc deficiency is an increased cancer risk

Accumulating evidence has recently indicated an association between zinc deficiency and cancers. Numerous epidemiological studies indicate that zinc deficiency contributes to increased cancer risk. It has been reported that zinc deficiency is correlated with increased tumor size, tumor stage, and increased unplanned hospitalizations in head and neck cancer patients^18^. A series of *in vivo* studies have been conducted by Fong and his colleagues to show that dietary zinc deficiency (ZD) increases the incidence of N-nitrosomethylbenzylamine (NMBA)-induced esophageal cancer^19–21^. High dietary zinc intake can also decrease the risk of colon cancer in a prospective cohort study^22^.

Several mechanisms contribute to the anti-tumor activity of zinc, including DNA damage, DNA repair, oxidative stress, immune function, and inflammation. Zinc deficiency can result in alterations in the oxidant defense system. Many proteins involved in the antioxidant defense system, including glutathione peroxidase, MTs, and Cu/Zn superoxide dismutase (SOD), require zinc for their activity. Zinc is also required by multiple proteins mediating DNA damages and repair responses. For example, p53 is an important zinc-containing transcription factor and is associated with the cellular response to DNA damage. Both *in vitro* and *in vivo* studies have indicated that zinc deficiency may cause oxidative DNA damage and compromise DNA damage repair responses^23,24^. Therefore, the impaired oxidant defense system, compromised DNA integrity, and damaged DNA repair enzymes increases the risk of cancer initiation and progression.

Zinc deficiency also causes dysfunction of the immune system. For innate immunity, zinc deficiency impairs the lytic activity of natural killer (NK) cells and phagocytosis of macrophages and neutrophils, and reduces cytokine production, whereas zinc supplementation has the opposite effect. For adaptive immunity, zinc deficiency induces thymic atrophy and lymphopenia, and compromises cell- and antibody-mediated immune responses. During T cell maturation in the thymus, zinc deficiency can lead to a 50% decrease in pre-T cells^25^. Zinc deficiency also decreases the production of Th1 cytokines (TNF-α, IL-2, and IFN-γ), whereas the Th2 cytokine response (IL-10, IL-6, and IL-4) is less affected. This change is accompanied by a Th1/Th2 functional imbalance, leading to the occurrence or progression of diseases in specific cancers^26^. Zinc deficiency also causes the loss of premature and immature B cells, and reduces antibody production. In addition, zinc deficiency can affect cytokine production and promote systemic inflammation. It has been demonstrated that zinc deficiency promotes the release of proinflammatory cytokines, such as TNF-α, IL-1β, IFN-γ, IL-2, IL-6, IL-8, S100a8, and S100a9^19^, which may be attributable to the ability of zinc to regulate nuclear factor-κB (NF-κB), the master regulator of inflammatory responses.

## Dysregulation of zinc metabolism in cancer

Altered zinc levels have been reported in the serum and malignant tissues of cancer patients. Many clinical studies use serum or plasma to estimate systemic zinc status as biomarkers of cancer patients. Epidemiological evidence has shown that the levels of serum zinc are strikingly reduced in most cancers, including cancers of the head and neck, breast, gastrointestinal tract, female genital tract, gallbladder, lung and bronchus, thyroid, leukemia, and esophageal squamous cell carcinoma (ESCC)^27–29^. Other studies measuring zinc levels in hair samples also detected decreased zinc levels in cancers of the lung, breast, and ovary^30,31^. However, melanoma patients have increased serum zinc levels^32^.

Zinc levels in malignant tissues are not always consistent with those in serum. Zinc levels are dramatically reduced in cancers of the prostate, pancreas, liver, gallbladder, cervix, and uterine myeloma. Although serum zinc levels are reduced, zinc levels are elevated in the cancerous tissues of several cancers, including cancers of the breast, lung, intestinal, and metastatic nasopharyngeal^28,29^. In a similar manner, conflicting results of zinc levels have also been described by several other studies in cancers of the prostate, breast, stomach, and lung^33,34^. The contradictory results in these studies may be attributed to the following reasons. First, all samples including hair, nails, urine, or plasma are susceptible to environmental influences, leading to an inaccurate estimation of zinc status in the observed populations. Additionally, the size of the samples and the experimental designs may also account for the mixed outcome results. Therefore, further large and well-designed cohort studies using standardized samples to estimate zinc status might elucidate the role of zinc in cancer.

Taken together, the present evidence suggests that zinc homeostasis is typically altered in a tissue-specific manner. The correlation between zinc levels and cancer progression appears to be complicated and less conclusive. The general opinion is that zinc shows antioxidant and proapoptotic properties by decreasing oxidative stress and improving immune function, which serves a protective effect on cancer initiation^35,36^. Notably, in some specific tumors such as breast cancer, mentioned above, the accumulating zinc levels are also observed in malignant tissues^29^. An explanation for this phenomenon is that increased demands for zinc are required for tumor survival and growth. The elevated zinc levels may facilitate the progression and malignancies of breast cancer.

## Zinc transporters and cancer

As described in previous sections, intracellular zinc concentration is regulated by zinc transporters, zinc-binding proteins, and the MTF1 zinc sensor. While the data for zinc status in tumor tissues are paradoxical, it has been widely observed that zinc dyshomeostasis in tumors results mainly from aberrant expression of zinc transporters, especially the ZIPs. The available data suggest that cellular zinc dyshomeostasis mediated by malfunctions of zinc transporters contributes to the development and progression of cancer. The present review mainly focuses on the roles of zinc transporters in breast, prostate, and pancreatic cancers, which have been thoroughly investigated in many previous studies.

### Prostate cancer

It is well recognized that zinc is important in maintaining prostate function and health. The human prostate gland accumulates excessive zinc because of the existence of specialized zinc-accumulating epithelial cells in the peripheral zone^37^. Zinc accumulation allows these cells to accumulate and secrete citrate in the prostatic fluid. The ZIP1 zinc transporter, located at the plasma membrane of normal acinar epithelial cells, is a functional transporter contributing to prostate zinc uptake and accumulation^38^.

Compelling clinical and experimental evidence consistently shows that zinc decrease is a hallmark characteristic of prostate cancer^39,40^. ZIP1 downregulation is also observed in prostate intraepithelial neoplasia and prostate adenocarcinoma, and is considered to be an important mechanism for the loss of zinc accumulation^41^. The overexpression of Ras-responsive element binding protein 1 (RREB1) is involved in ZIP1 downregulation in prostate cancer. RREB1 is a downstream effector of the Ras-Raf-MEK-ERK pathway and is upregulated during prostate cancer progression^42,43^. Upregulation of RREB1 in the early development of malignancy leads to ZIP1 downregulation and a subsequent zinc decrease in prostate cancer. Other zinc importers, such as ZIP2, ZIP3 and ZnT4 also show decreased expression in prostate cancer^44,45^.

**Figure 3:**
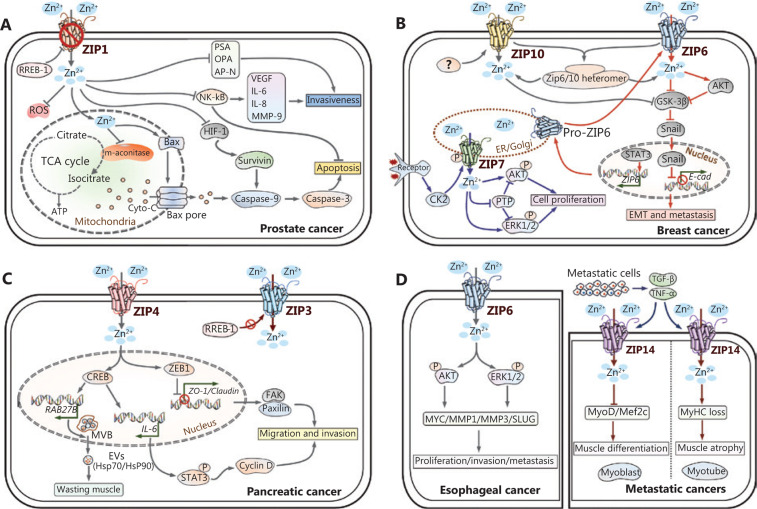
Dysregulation of zinc signaling pathways in different cancers. The transporter-mediated imbalance of intracellular zinc can contribute to the development and progression of cancer, including prostate cancer (A), breast cancer (B), pancreatic cancer (C), esophageal cancer, and metastatic cancers (D). Zinc fluctuations contribute to the disturbance of certain signaling pathways involved in the malignant properties of cancer cells. For clarity, blue arrows are used to indicate ZIP7-mediated pathways, and red arrows are used to represent ZIP6-mediated pathways in breast cancer.

Increasing studies show that zinc affects the apoptosis and metabolism of prostate cells. The mechanisms include suppression of mitochondrial aconitase activity and citrate oxidation^46,47^, induction of mitochondrial apoptogenesis^48^, an increased Bax/Bcl-2 ratio^49^, induction of HIF-1α degradation, and a decreased expression of survivin^50^. In addition, zinc has been shown to suppress the metastatic potential of prostate cancer by inhibiting NF-κB signaling^51,52^, and suppressing the invasive potential of the proteolytic enzyme urokinase-type plasminogen activator, aminopeptidase N, and prostate specific antigen (PSA)^53,54^. Based on these observations, zinc supplementation may be an effective therapy for prostate cancer (**Figure 3A**).

In contrast, studies also show that zinc can promote growth and invasion of prostate cancer cells by increasing telomerase activity or suppressing the anti-tumor potential of bisphosphonates^55^. Further studies by Wong et al.^56^ have shown that high zinc supplementation may have inhibitory effects on prostate cancer cell growth, while continuous exposure results in more aggressive behavior in cancer cells.

Given that zinc plays diverse roles in cell signaling, more studies investigating the mechanisms underlying the maintenance of zinc homeostasis are required to fully define its role in prostate carcinogenesis.

### Breast cancer

Zinc is also critical for normal mammary gland expansion, remodeling, and lactation^37^. In mammary epithelial cells, numerous zinc importers and exporters function coordinately to maintain zinc homeostasis.

Studies have recently examined aberrant zinc homeostasis during the initiation and progression of breast cancer^28,29^. Regardless of the decrease in serum zinc previously mentioned, a significantly increased zinc level in breast cancer tissues has been consistently observed^57^. *In vivo* studies using a N-methyl-N-nitrosourea (MNU)-induced rat mammary carcinogenesis model have also demonstrated higher zinc accumulation in malignant tissue than in the normal mammary gland, consistent with the findings reported in human breast cancer^58,59^.

Notably, the zinc transporting network shows a distinct subtype-specific dysregulation in breast cancer^15^. X-ray analysis of malignant tumors showed high zinc accumulation around the luminal tumor periphery, while zinc was distributed evenly in basal tumors. In addition, gene expression profiling of the zinc transporting network was significantly different in these two subtypes. Numerous zinc transporters show high expression in luminal cells, except for MTs, ZIP10, and ZnT1. The changes in zinc transporters are also in accordance with subtype-specific alterations in the subcellular zinc pools. For example, ZnT2 is strikingly overexpressed in luminal cells (T47D, poorly invasive) and luminal tumors, which could protect cells from zinc cytotoxicity by mobilizing zinc into intracellular vesicles^60^. However, the loss of ZnT2 expression in basal-like cells (MDA-MB-231, highly invasive) and basal tumors leads to zinc accumulation and ultimately contributes to the invasive malignant phenotype. This subtype-specific zinc accumulation in intracellular pools may explain the phenotypic differences of malignant breast cancers and help develop novel diagnostic and therapeutic methods.

For the zinc transporters in breast cancer, ZIP6 was first identified as an estrogen-regulated gene and positively correlated with estrogen receptor (ER). In addition, high ZIP6 expression has been proposed to be a reliable marker of the luminal A subtype of breast cancer^61^. ZIP6 has been shown to play a mechanistic role in modulating the epithelial-mesenchymal transition (EMT) in breast cancer. ZIP6 is transactivated by STAT3 during gastrulation in zebrafish. Then Snail, a zinc-finger transcription factor, translocates to the nucleus and represses the expression of the epithelial adhesion molecule, E-cadherin^62^. The overexpression of ZIP6 in breast cell lines and tumors shows a strongly positive correlation with phosphorylated (activated) STAT3^63^. Hogstrand et al.^64^ demonstrated that ZIP6 is transcriptionally induced by STAT3 and activated by N-terminal cleavage. ZIP6 then translocates to the plasma membrane and promotes the accumulation of cellular zinc. A zinc influx/GSK-3β inhibition/Snail activation/E-cadherin loss pathway is sequentially activated, resulting in cell migration and metastasis (**Figure 3B**).

ZIP10 also contributes to the invasive behavior of breast cancer^65,66^. High mRNA expression of *ZIP10* is associated with lymph node metastasis. Elevated *ZIP10* mRNA levels are also observed in highly invasive breast cancer cell lines, including MDA-MB-435S and MDA-MB-231. ZIP10-mediated zinc uptake is required for the malignant behavior of breast cancer cells, as *ZIP10* gene attenuation or intracellular zinc depletion by the zinc chelator TPEN inhibits the migration of MDA-MB-231 cells. Furthermore, ZIP10 may form a functional heteromeric complex with ZIP6. These molecules have been found to regulate embryonic development and cell migration by inactivating GSK-3 and downregulating E-cadherin. These results indicate that zinc transporters may integrate to conduct biological activities, and the results further highlight the important roles of zinc transporters in tumorigenesis (**Figure 3B**).

Recently, ZIP7 has also been shown to be involved in aberrant growth factor signaling in breast cancer cells^67^. Increased ZIP7 expression has been shown to contribute to zinc accumulation in tamoxifen-resistant (TamR) breast cancer cells. ZIP7 is phosphorylated by the protein kinase CK2 at the Ser275 and Ser276 residues^68^. Then, zinc release from the ER and Golgi apparatus results in the activation of the downstream signaling pathway and promotes tumor growth and invasion^69^. Moreover, recent findings suggest that ZIP7 is essential to maintain intestinal epithelial homeostasis and skin dermis development^70,71^ (**Figure 3B**).

Other zinc modulators including ZIP9 and MTs have also been implicated in breast cancer progression. ZIP9 acts as a novel membrane androgen receptor in both breast cancer and prostate cancer cell lines. ZIP9 mediates testosterone promotion of apoptosis through MAP kinase- and zinc-dependent pathways^72^. MT overexpression is associated with chemoresistance in patients who received adjuvant therapy after surgery, and promotes breast cancer cell invasion by increasing the expression of matrix metalloproteinase-9 (MMP9)^73,74^.

### Pancreatic cancer

The pancreas functions as both an endocrine and exocrine gland. The exocrine gland produces enzymes required for digestion, while the endocrine gland produces hormones, especially insulin, to control metabolism. Zinc is involved in many processes within the pancreas, and cellular zinc dysregulation could be correlated with the pancreatic cancer development and progression^75^. However, the function of zinc and zinc transporters in pancreatic cancer is controversial.

In situ zinc staining shows that zinc levels in pancreatic ductal and acinar epithelial cells are strikingly reduced in premalignant PanIN lesions as well as during the development of pancreatic cancer^76^. ZIP3, localized predominantly at the basilar membrane in normal ductal/acinar epithelium, is the likely zinc transporter for cellular zinc uptake and accumulation. Downregulation of ZIP3 as well as Ras responsive binding protein (RREB1) occur simultaneously with zinc loss during the progression to malignancy^77^. These changes are considered to be a common and early event in pancreatic cancer progression, which likely facilitate malignant cells to eliminate the cytotoxic effects of zinc. Although the expression levels of ZIP1 and ZIP2 are also decreased, they are not responsible for zinc loss in pancreatic adenocarcinoma^76^ (**Figure 3C**).

However, studies by Li et al.^78,79^ reported that another zinc transporter, ZIP4, localized at the basolateral membrane of pancreatic β cells, is markedly upregulated in cancerous tissues compared with adjacent normal tissues. Overexpression of ZIP4 enhances zinc accumulation and cell growth in both cell lines and tumorigenic animal models. The gene expression profile conducted by Yang et al.^80^ revealed that, almost all zinc transporters except for ZIP4, are decreased in malignant pancreatic tissues compared to non-malignant tissues. This finding is in agreement with the previous results that ZIP4 might contribute to the cellular zinc accumulation of pancreatic cancer. The underlying mechanisms for ZIP4 involvement in the proliferation and metastasis of pancreatic cancer can be multifaceted. These mechanisms include the activation of the zinc finger transcription factor CREB-mediated IL-6/STAT3/cyclin D1 pathway, the overexpression of neuropilin-1 (NRP-1), vascular endothelial growth factor (VEGF), and matrix metalloproteases (MMP-2 and MMP-9), repression of zona occludens-1 (ZO-1) and claudin-1 by zinc finger E-box binding homeobox 1 (ZEB1), and RAB27B-mediated release of extracellular vesicles from cancer cells^81–83^ (**Figure 3C**).

## Zinc signaling in other cancers

There are numerous studies reporting aberrant zinc status and zinc transporter expression in other cancers. In our previous study, we characterized the mechanism of ZIP6 overexpression in ESCC, indicating that targeted inhibition of ZIP6 or modulation of intracellular zinc homeostasis might be effective for the treatment of ESCC^84^ (**Figure 3D**). In addition, ZIP14 downregulation is associated with decreased zinc levels in HCC^85,86^. However, recent studies have identified ZIP14 as a critical mediator of cachexia development in metastatic cancer models^87,88^ (**Figure 3D**). Zinc transporters involved in other cancers, such as ovarian cancer, renal cell carcinoma (RCC), cervical cancer, oral squamous cell carcinoma (OSCC), nasopharyngeal carcinoma (NPC), and lung cancer are also briefly summarized in **Table 1**^89–108^.

## Clinical applications of zinc and zinc transporters

As previously described, epidemiological studies provide compelling evidence that zinc deficiency is associated with increased cancer risk, suggesting that zinc might be utilized in the prevention and treatment of malignancy.

An *in vitro* study showed that exogenous zinc could increase protein ubiquitination and lead to the cell death of pancreatic cancer cells^109^. Treatment of pancreatic cancer cells with zinc in the presence of the ionophore compound, pyrrolidine dithiocarbamate, induces cellular zinc accumulation and leads to caspase-independent apoptosis *via* reactive oxygen species (ROS)/mitochondrial apoptosis inducing factor (AIF)^110^. These studies indicate that zinc can be used to treat pancreatic cancer. *In vivo* studies have also indicated that zinc replenishment in NMBA-treated rodent models substantially reduces the development or progression of esophageal cancer^111,112^. Furthermore, animal cancer models reported that zinc supplementation may prevent the development of colon cancer^113^. Zinc supplementation is also effective in improving local recurrence-free survival in patients with advanced nasopharyngeal carcinoma and head and neck cancers^114^.

Zinc has also been shown to be useful in the prevention and treatment of prostate cancer. Ghosh et al.^115^ reported that local zinc depletion is associated with a higher Gleason score in prostatic malignancy, which may improve the selection of patients for biopsy, biopsy site selection, and local therapy. In addition, prostate cancer progression could be imaged *in vivo* by detecting decreased zinc levels in a transgenic adenocarcinoma of the mouse prostate (TRAMP) model, using a novel zinc fluorescent sensor (ZPP1). Because the downregulation of ZIP1 and loss of zinc occur prior to discernible histopathological abnormalities in premalignancy, zinc status could serve as an auxiliary means of PSA screening.

Nevertheless, it should be mentioned that the results of studies investigating the therapeutic effect of zinc are controversial in prostate cancer. An *in vivo* study showed that overexpression of ZIP1 transporter led to enhanced zinc uptake and reduced tumor growth in a xenograft model^116^. Zinc administration inhibits PC3 tumor growth by inducing apoptosis^117^. Zinc at optimal levels can also be protective against prostate carcinogenesis in TRAMP mice and in N-methyl-N-nitrosourea/testosterone-induced prostate cancer in rats^118^. Moreover, direct intratumoral injection of zinc inhibits the growth of prostate cancer and substantially prolongs animal survival with almost no detectable cytotoxicity to other tissues^119^. However, another study found that high dietary zinc supplementation leads to zinc accumulation in the prostate and induces prostate intraepithelial neoplasia in a murine model of prostate cancer^120^.

These conflicting results may be partly due to the discrepancies in the experimental design, the amount or duration of zinc administered, and the method to determine plasma/serum zinc status. In addition, zinc accumulation might fail due to the diminished expression of zinc importers despite increased dietary intake. Although zinc might be a potential dietary chemopreventive or chemotherapeutic agent in some types of cancers^121^, dietary supplementation with zinc has issues with bioavailability and bioactivity. Consequently, zinc as a pharmacological agent is complex and requires further development.

However, studies focusing on utilizing zinc-related proteins in the treatment of cancer may be more relevant. Because the zinc LIV-1(SLC39A6) transporter is expressed in all breast cancer subtypes, Seattle Genetics constructed a novel antibody-drug conjugate, SGN-LIV1A, targeting LIV-1 for the treatment of metastatic breast cancer^122^. SGN-LIV1A is currently in a Phase 1 trial and may be a new therapy for patients with metastatic breast cancer and cancers with LIV-1-positive indications. In addition, NVS-ZP7-4 is also recently identified as a ZIP7 inhibitor^123^. This chemical may be a potential treatment of cancer patients with high ZIP7 expression.

## Conclusions and perspectives

**Figure 4:**
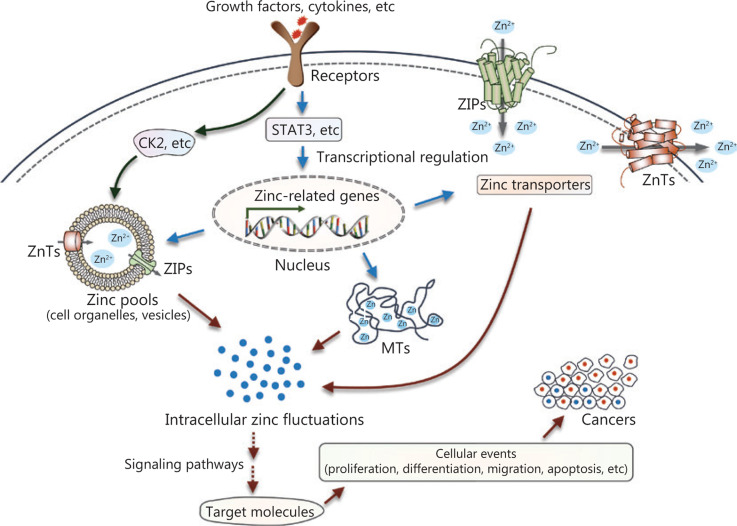
Summary of zinc signaling in pathogenesis. Intracellular zinc fluctuations can be triggered by zinc release from intracellular stores, or transcriptional regulation of proteins required for zinc metabolism and homeostasis. Then, zinc modulates multiple zinc-requiring proteins or phosphorylation-dependent signaling cascades, which contribute to numerous cellular events, such as survival, differentiation, proliferation, and migration, ultimately causing the initiation or progression of cancer.

Over the past few decades, accumulating evidence has revealed disturbances of zinc metabolism and homeostasis in cancer (**Figure 4**). Extracellular stimuli such as growth factors and cytokines, which are probably produced in the microenvironments surrounding cancerous tissue, can directly or indirectly affect intracellular zinc status. In one case, an extracellular stimulus could activate zinc transporters on the intracellular zinc stores, such as ZIP7 on the ER through phosphorylation at specific residues by the protein kinase, CK2, resulting in cytosolic free zinc. The increased intracellular free zinc functions as an intracellular second messenger and leads to the phosphorylation of ERK1/2 and AKT, and cell migration. In another case, extracellular stimuli can modulate various signaling cascades, such as the extensively studied JAK-STAT signaling pathway. These processes contribute to transcriptional regulation of proteins involved in zinc uptake, distribution, storage, and release. The dysregulation of intracellular zinc homeostasis then influences multiple zinc-requiring proteins and phosphorylation-dependent signaling cascades (for example, MAPKs, Akt, protein tyrosine phosphatases, MMPs, and zinc-finger proteins), thereby playing important roles in cell development, proliferation, and cell death. Because altered zinc homeostasis is tissue-specific, further studies are required to elucidate the exact role of zinc in specific cancers. Zinc sensors for quantitative detection of zinc levels or targeting specific subcellular zinc pools may provide more precise information regarding the role of zinc dyshomeostasis in the development and progression of cancer^124,125^. In addition, chemicals or treatments that specifically modulate zinc-transporter functions or zinc levels in subcellular zinc pools may serve as effective tools to treat patients with cancer.

## Figures and Tables

**Table 1:** Zinc dysregulation in cancers

Cancers	Serum	Tissue	Aberrant transporter	References
Prostate cancer	Contradiction^†^	Decreased	ZIP1, ZIP2, ZIP3, ZIP4, ZIP9, ZnT4	^34,41,43–45^
Breast cancer	Decreased	Increased	ZIP6, ZIP7, ZIP9, ZIP10, ZnT2	^31,33,60,65,67^
Pancreatic cancer	NR^‡^	Decreased	ZIP3, ZIP4	^77,79^
Hepatocellular cancer (HCC)	Decreased	Decreased	ZIP4, ZIP14, ZnT9	^86,89,90^
Esophageal squamous cell carcinoma (ESCC)	Decreased	Decreased	ZIP5, ZIP6	^11,91,92^
Ovarian cancer	Decreased	Decreased	ZIP4	^31,93^
Cervical cancer	Decreased	Decreased	ZIP7	^94,95^
Kidney cancer	NR	Decreased	ZIP1, ZIP10	^96–98^
Gastric cancer	Decreased	Increased	Contradiction^†^	^99,100^
Lung cancer	Decreased	Increased	ZIP4	^101–103^
Bladder cancer	Decreased	NR	ZIP11, ZnT1	^95,104,105^
Oral squamous cell carcinoma (OSCC)	NR	NR	ZIP4	^106^
Nasopharyngeal carcinoma (NPC)	NR	Increased	ZIP4	^107,108^

^†^Conflicting results are reported in several articles. ^‡^Not reported.
