# Supplementary material for: Zinc dysregulation in cancers and its potential as a therapeutic target
Source: Cancer Biol Med. 2020 Aug 15;17(3):612–25. doi: 10.20892/j.issn.2095-3941.2020.0106 (PMC7476080; doi:10.20892/j.issn.2095-3941.2020.0106)
Supplement: Supplementary file 1 [file cbm-17-612-s001.pdf]

Supplementary materials

Table S1 Genetic mutation studies in mice and human

Gene	Alias	Mutation type	Phenotype and disorders in mouse and human	Reference
SLC39A1	ZIP1	KO	Abnormal embryonic development in zinc-limiting condition	1
SLC39A2	ZIP2	KO	Abnormal embryonic development in zinc-limiting condition	2
SLC39A3	ZIP3	KO	Abnormal embryonic and T-cell development in zinc-limiting condition	3
SLC39A4	ZIP4	KO<br>Intestine-specific KO<br>Mutation	Embryonic lethal<br>Disruption of stem cell niche and intestine integrity<br>Acrodermatitis enteropathica (AE)	4-7
SLC39A5	ZIP5	KO<br><br>Intestine-specific KO<br>Pancreas-specific KO<br><br>Mutation	Increased hepatic zinc in zinc-adequate condition and impaired accumulation of pancreatic zinc in zinc-excess conditions<br>Increased accumulation of pancreatic zinc in zinc-adequate conditions<br>Modest impairment of pancreatic zinc retention in zinc-adequate conditions<br>Autosome dominant non-syndromic high myopia	8,9
SLC39A6	ZIP6, LIV1	Not reported		
SLC39A7	ZIP7, KE4	Intestine-specific KO<br>Connective tissues-specific KO	Disruption of colon epithelial cell differentiation and proliferation<br>Dermal dysplasia due to accumulated zinc in the endoplasmic reticulum	10,11
SLC39A8	ZIP8	Chondrocyte-specific KO<br>Hypomorphic mutation	Suppression of surgically induced osteoarthritis pathogenesis<br>Hypoplasia of multiple organs and defects in hematopoiesis<br>Abnormal innate immune function	12-15
SLC39A9	ZIP9	Not Reported		
SLC39A10	ZIP10	Epidermis-specific KO<br>Thymus-specific KO<br>B cell-specific KO	Significant epidermal hypoplasia<br>Hypoplastic thymus<br>Abnormal early B-cell development<br>Impaired humoral immune response	16-18
SLC39A11	ZIP11	Not reported		
SLC39A12	ZIP12	KO (Rat)	Attenuation of pulmonary hypertension in a hypoxic atmosphere	19
SLC39A13	ZIP13	KO<br>Mutation	Connective tissue dysplasia<br>Spondylocheiro dysplastic Ehlers-Danlos syndrome (SCD-EDS)	20,21
SLC39A14	ZIP14	KO<br><br><br>Mutation	Growth retardation and impaired gluconeogenesis<br>Dampened insulin signaling, hypertrophied adipocytes, and increased adipose cytokine production and plasma leptin<br>Impaired hepatocyte proliferation during liver regeneration after hepatectomy<br>Hypermanganesemia with dystonia 2, Childhood-onset Parkinsonism-dystonia	22-28
SLC30A1	ZnT1	KO	Embryonic lethal	29
SLC30A2	ZnT2	Mutation	Low zinc in milk	30-32
SLC30A3	ZnT3	KO	Prone to seizures<br>Alzheimer's disease-like abnormalities<br>Required for hippocampus dependent memory	33-35
SLC30A4	ZnT4	Mutation	Low zinc in milk	36

Table S1 Continued

Gene	Alias	Mutation type	Phenotype and disorders in mouse and human	Reference
SLC30A5	ZnT5	KO	Growth retardation, osteopenia, male-specific cardiac death<br>Impaired mast cell functions	37,38
SLC30A6	ZnT6	Not reported		
SLC30A7	ZnT7	KO	Growth retardation, low body zinc accumulation and low fat accumulation<br>Susceptible to diet-induced glucose intolerance and insulin resistance	39,40
SLC30A8	ZnT8	KO	Impairment of insulin secretion and insulin-crystal formation; type 2 diabetes mellitus	41-45
		SNP	Type 1 and 2 diabetes mellitus	
SLC30A9	ZnT9	Not reported		
SLC30A10	ZnT10	Mutation	Parkinsonism, dystonia, hypermanganesemia, polycythemia, chronic liver Disease	46,47

KO, knockout.

References

1. Duffer-Beattie J, Huang ZL, Geisser J, Xu W, Andrews GK. Mouse ZIP1 and ZIP3 genes together are essential for adaptation to dietary zinc deficiency during pregnancy. Genesis. 2006; 44: 239-51.
2. Peters JL, Dufner-Beattie J, Xu W, Geiser J, Lahner B, Salt DE, et al. Targeting of the mouse SLC39A2 (ZIP2) gene reveals highly cell-specific patterns of expression, and unique functions in zinc, iron, and calcium homeostasis. Genesis. 2007; 45: 339-52.
3. Duffer-Beattie J, Huang ZL, Geisser J, Xu W, Andrews GK. Generation and characterization of mice lacking the zinc uptake transporter ZIP3. Mol Cell Biol. 2005; 25: 5607-15.
4. Kory S, Drano B, Bibeau S, Grauwet S, Khari M, Hamoun R, et al. Identification of SLC39A4, a gene involved in Acrodermatitis enteropathica. Nat Genet. 2002; 31: 239-40.
5. Dufner-Beattie J, Weaver BP, Geiser J, Bilgen M, Larson M, Xu W, et al. The mouse Acrodermatitis enteropathica gene SLC39A4 (ZIP4) is essential for early development and heterozygosity causes hypersensitivity to zinc deficiency. Hum Mol Genet. 2007; 16: 1391-9.
6. Bin BH, Bhin J, Kim NH, Lee SH, Jung HS, Seo J, et al. An Acrodermatitis enteropathica-associated ZN transporter, ZIP4, regulates human epidermal homeostasis. J Invest Dermatol. 2017; 137: 874-83.
7. Geiser J, Venken KJ, De Lisle RC, Andrews GK. A mouse model of Acrodermatitis enteropathica: loss of intestine zinc transporter ZIP4 (SLC39A4) disrupts the stem cell niche and intestine integrity. PLoS Genet. 2012; 8: e1002766.
8. Geisser J, De Lisle RC, Andrews GK. The zinc transporter ZIP5 (SLC39A5) regulates intestinal zinc excretion and protects the pancreas against zinc toxicity. PLoS One. 2013; 8: e82149.
9. Guo H, Jin X, Zhu T, Wang T, Tong P, Tian L, et al. SLC39A5 mutations interfering with the BMP/TGF-beta pathway in non-syndromic high myopia. J Med Genet. 2014; 51: 518-25.
10. Ohashi W, Kimura S, Iwanaga T, Furusawa Y, Irie T, Izumi H, et al. Zinc transporter SLC39A7/ZIP7 promotes intestinal epithelial self-renewal by resolving ER stress. PLoS Genet. 2016; 12: e1006349.
11. Bin BH, Bhin J, Seo J, Kim SY, Lee E, Park K, et al. Requirement of zinc transporter SLC39A7/ZIP7 for dermal development to fine-tune endoplasmic reticulum function by regulating protein disulfide isomerase. J Invest Dermatol. 2017; 137: 1682-91.
12. Kim JH, Jeon J, Shin M, Won Y, Lee M, Kwak JS, et al. Regulation of the catabolic cascade in osteoarthritis by the zinc-ZIP8-MTF1. Cell. 2014; 156: 730-43.
13. Liu MJ, Bao S, Galvez-Peralta M, Pyle CJ, Radinsky AC, Pavlovich RE, et al. ZIP8 regulates host defense through zinc-mediated inhibition of NF-κB. Cell Rep. 2013; 3: 386-400.
14. Galvez-Peralta M, He L, Jorge-Nobert LF, Wang B, Miller ML, Expert BL, et al. ZIP8 zinc transporter: indispensable role for both multiple-organ organogenesis and hematopoiesis in utero. PLoS One. 2012; 7: e36055.
15. Pickerel JK, Brisa T, Liu JZ, Securely L, Tung JY, Hinds DA. Detection and interpretation of shared genetic influences on 42 human traits. Nat Genet. 2016; 48: 709-17.
16. Bin BH, Bhin J, Takaishi M, Toyoshima KE, Kalamata S, Ito K, et al. Requirement of zinc transporter ZIP10 for epidermal development: implication of the ZIP10-p63 axis in epithelial homeostasis. Proc Natl Acad Sci U S A. 2017; 114: 12243-48.
17. Miya T, Hojyo S, Aikawa T, Kawamura M, Irie T, Ogura H, et al. Zinc transporter SLC39A10/ZIP10 facilitates ant apoptotic signaling during early B-cell development. Proc Natl Acad Sci U S A. 2014; 111: 11780-5.
18. Hojyo S, Miya T, Fujihara H, Kawamura M, Yasuda T, Hijikata A, et al. Zinc transporter SLC39A10/ZIP10 controls humoral

immunity by modulating B-cell receptor signal strength. Proc Natl Acad Sci U S A. 2014; 111: 11786-91.
19. Zhao L, Oliver E, Maratou K, Atanur SS, Dubois OD, Cotroneo E, et al. The zinc transporter ZIP12 regulates the pulmonary vascular response to chronic hypoxia. Nature. 2015; 524: 356-60.
20. Fukada T, Civic N, Furuichi T, Shimoda S, Mishima K, Higashiyama H, et al. The zinc transporter SLC39A13/ZIP13 is required for connective tissue development; its involvement in BMP/TGF-beta signaling pathways. PLoS One. 2008; 3: e3642.
21. Giunta C, Elcioglu NH, Albrecht B, Eich G, Chambaz C, Janecke AR, et al. Spondylocheiro dysplastic form of the Ehlers-Danlos syndrome – an autosomal-recessive entity caused by mutations in the zinc transporter gene SLC39A13. Am J Hum Genet. 2008; 82: 1290-305.
22. Hojyo S, Fukada T, Shimoda S, Ohashi W, Bin BH, Koseki H, et al. The zinc transporter SLC39A14/ZIP14 controls G-protein coupled receptor-mediated signaling required for systemic growth. PLoS One. 2011; 6: e18059.
23. Tuschl K, Meyer E, Valdivia LE, Zhao N, Dadswell C, Abdul-Sada A, et al. Mutations in SLC39A14 disrupt manganese homeostasis and cause childhood-onset parkinsonism-dystonia. Nat Commun. 2016; 7: 11601.
24. Troche C, Aydemir TB, Cousins RJ. Zinc transporter SLC39A14 regulates inflammatory signaling associated with hypertrophic adiposity. Am J Physiol Endocrinol Metab. 2016; 310: E258-68.
25. Aydemir TB, Chang SM, Guthrie GJ, Maki AB, Ryu MS, Karabiyik A, et al. Zinc transporter ZIP14 functions in hepatic zinc, iron and glucose homeostasis during the innate immune response (endotoxemia). PLoS One. 2012; 7: e48679.
26. Aydemir TB, Troche C, Kim MH, Cousins RJ. Hepatic ZIP14-mediated zinc transport contributes to endosomal insulin receptor trafficking and glucose metabolism. J Biol Chem. 2016; 291: 23939-51.
27. Kim MH, Aydemir TB, Kim J, Cousins RJ. Hepatic ZIP14-mediated zinc transport is required for adaptation to endoplasmic reticulum stress. Proc Natl Acad Sci U S A. 2017; 114: E5805-14.
28. Aydemir TB, Sitren HS, Cousins RJ. The zinc transporter ZIP14 influences c-Met phosphorylation and hepatocyte proliferation during liver regeneration in mice. Gastroenterology. 2012; 142: 1536-46 e5.
29. Andrews GK, Wang H, Dey SK, Palmiter RD. Mouse zinc transporter 1 gene provides an essential function during early embryonic development. Genesis. 2004; 40: 74-81.
30. Chowanadisai W, Lonnerdal B, Kelleher SL. Identification of a mutation in SLC39A2 (ZnT-2) in women with low milk zinc concentration that results in transient neonatal zinc deficiency. J Biol Chem. 2006; 281: 39699-707.
31. Lee S, Hennigar SR, Alam S, Nishida K, Kelleher SL. Essential role for zinc transporter 2 (ZnT-2)-mediated zinc transport in mammary gland development and function during lactation. J Biol Chem. 2015; 290: 13064-78.
32. Itsumura N, Kibihara Y, Fukue K, Miyata A, Fukushima K, Tamagawa-Mineoka R, et al. Novel mutations in SLC39A2 involved in the pathogenesis of transient neonatal zinc deficiency. Pediatr Res. 2016; 80: 586-94.
33. Cole TB, Robbins CA, Wenzel HJ, Schwartzkroin PA, Palmiter RD. Seizures and neuronal damage in mice lacking vesicular zinc. Epilepsy Res. 2000; 39: 153-69.
34. Sindreu C, Palmiter RD, Storm DR. Zinc transporter ZnT-3 regulates presynaptic Erk1/2 signaling and hippocampus-dependent memory. Proc Natl Acad Sci U S A. 2011; 108: 3366-70.
35. Adlard PA, Parncutt JM, Finkelstein DI, Bush AI. Cognitive loss in zinc transporter-3 knock-out mice: a phenocopy for the synaptic and memory deficits of Alzheimer's disease? J Neurosci. 2010; 30: 1631-6.
36. Huang L, Gitschier J. A novel gene involved in zinc transport is deficient in the lethal milk mouse. Nat Genet. 1997; 17: 292-7.
37. Inoue K, Matsuda K, Itoh M, Kawaguchi H, Tomoike H, Aoyagi T, et al. Osteopenia and male-specific sudden cardiac death in mice lacking a zinc transporter gene, ZnT5. Hum Mol Genet. 2002; 11: 1775-84.
38. Nishida K, Hasegawa A, Nakae S, Oboki K, Saito H, Yamasaki S, et al. Zinc transporter ZnT5/SLC30A5 is required for the mast cell-mediated delayed-type allergic reaction but not the immediate-type reaction. J Exp Med. 2009; 206: 1351-64.
39. Huang L, Yu YY, Kirschke CP, Gertz ER, Lloyd KK. ZnT7 (SLC30A7)-deficient mice display reduced body zinc status and body fat accumulation. J Biol Chem. 2007; 282: 37053-63.
40. Huang L, Kirschke CP, Lay YA, Levy LB, Lamirande DE, Zhang PH. ZnT7-null mice are more susceptible to diet-induced glucose intolerance and insulin resistance. J Biol Chem. 2012; 287: 33883-96.
41. Tamaki M, Fujitani Y, Hara A, Uchida T, Tamura Y, Takeno K, et al. The diabetes-susceptible gene SLC30A8/ZnT8 regulates hepatic insulin clearance. J Clin Invest. 2013; 123: 4513-24.
42. Lemaire K, Ravier MA, Schraenen A, Creemers JW, Van de Plas R, Granvik M, et al. Insulin crystallization depends on zinc transporter ZnT8 expression, but is not required for normal glucose homeostasis in mice. Proc Natl Acad Sci U S A. 2009; 106: 14872-7.
43. Sladek R, Rocheleau G, Rung J, Dina C, Shen L, Serre D, et al. A genome-wide association study identifies novel risk loci for type 2 diabetes. Nature. 2007; 445: 881-5.
44. Wenzlau JM, Moua O, Sarkar SA, Yu L, Rewers M, Eisenbarth GS, et al. SlC30A8 is a major target of humoral autoimmunity in type 1 diabetes and a predictive marker in prediabetes. Ann N Y Acad Sci. 2008; 1150: 256-9.
45. Nicolson TJ, Bellomo EA, Wijesekara N, Loder MK, Baldwin JM, Gyulkhandanyan AV, et al. Insulin storage and glucose homeostasis in mice null for the granule zinc transporter ZnT8 and studies of the type 2 diabetes-associated variants. Diabetes. 2009; 58: 2070-83.
46. Quadri M, Federico A, Zhao T, Breedveld GJ, Battisti C, Delnooz C, et al. Mutations in SLC39A10 cause parkinsonism and dystonia with hypermanganesemia, polycythemia, and chronic liver disease. Am J Hum Genet. 2012; 90: 467-77.
47. Tuschl K, Clayton PT, Gospe Jr SM, Gulab S, Ibrahim S, Singhi P, et al. Syndrome of hepatic cirrhosis, dystonia, polycythemia, and hypermanganesemia caused by mutations in SLC39A10, a manganese transporter in man. Am J Hum Genet. 2012; 90: 457-66.
